# Epstein–Barr virus-associated inflammatory polyp of the proximal cervical trachea causing central airway obstruction: a case report

**DOI:** 10.3389/fmed.2026.1940485

**Published:** 2026-09-03

**Authors:** Ziran Lei, Ning Zheng, Linlin Wang, Ling Fang, Xiang Wu

**Affiliations:** 1 Department of Respiratory, Huangshan Huaze Hospital of Integrated Traditional Chinese and Western Medicine, Huangshan, China; 2 Department of Medicine, Dinfectome Inc., Nanjing, China

**Keywords:** central airway obstruction, EBER, Epstein–Barr virus, targeted next-generation sequencing, tracheal inflammatory polyp

## Abstract

**Background:**

Intraluminal lesions of the central airway can cause severe obstruction and are usually malignant. Epstein–Barr virus (EBV)-associated inflammatory tracheal polyps are extremely rare and may mimic airway tumors or EBV-associated lymphoproliferative disorders.

**Case presentation:**

A 35-year-old woman presented with progressive dyspnea and wheezing for 1 month. Contrast-enhanced chest computed tomography and bronchoscopy revealed a solitary intraluminal polypoid lesion arising from the left anterior wall of the proximal cervical trachea, without true subglottic involvement. The lesion extended from approximately 26.3 mm to 40.8 mm caudal to the vocal cords, with a craniocaudal length of approximately 14.5 mm. The minimum residual airway diameter at the narrowest point was approximately 1.7 mm, and the estimated luminal obstruction was approximately 54%. Emergency rigid bronchoscopic cryoresection and cryotherapy were performed, resulting in rapid symptom relief. Histopathology showed polypoid tissue fragments from the proximal cervical trachea with reactive lymphoid hyperplasia beneath the squamous epithelium. Immunohistochemistry supported a reactive process, with no evidence of lymphoma or malignancy. EBER *in situ* hybridization was positive in a subset of lymphocytes. Tissue-based targeted next-generation sequencing detected abundant EBV sequences, which were confirmed by PCR and Sanger sequencing. The final diagnosis was EBV-associated inflammatory polyp of the proximal cervical trachea.

**Conclusions:**

EBV-associated inflammatory tracheal polyps may present as life-threatening airway obstruction. For rare airway lesions with reactive hyperplasia and EBER positivity, integrated histopathological, immunohistochemical, *in situ* hybridization, and molecular analyses are important to avoid misdiagnosis. Bronchoscopic intervention can effectively relieve obstruction, but follow-up is needed to monitor recurrence and airway stenosis.

## Introduction

Space-occupying lesions of the large airways can cause progressive dyspnea, wheezing/stridor, or laryngeal stridor, and in severe cases may rapidly progress to life-threatening central airway obstruction. Girvin et al. conducted a systematic review of the radiological, bronchoscopic, and pathological features of tracheobronchial space-occupying lesions, reporting that approximately 85% of these lesions are malignant and 15% are benign. Among malignant lesions, primary pulmonary malignancies account for approximately 75%, whereas benign lesions are predominantly inflammatory, infectious, or granulomatous, comprising approximately 65% of benign cases (1). Therefore, when an intratracheal lesion causes progressive airway compromise, timely relief of obstruction and definitive etiological diagnosis are equally important.

Epstein–Barr virus (EBV), also known as human herpesvirus 4 (HHV-4), belongs to the gammaherpesvirus subfamily and is a double-stranded DNA virus that is widely prevalent in the human population (2). More than 95% of adults worldwide have evidence of EBV infection, and most infected individuals remain asymptomatic (3). The clinical manifestations of EBV infection are influenced by age and immune status: primary infection in children or adolescents may present as infectious mononucleosis, whereas immunocompromised individuals are more likely to develop nonspecific or atypical manifestations (4). EBV primarily infects B lymphocytes and establishes lifelong latency in memory B cells. Its infection cycle generally includes primary infection, latency, and reactivation, and is regulated by the interaction between the virus and host immune responses (5). Antibodies against viral capsid antigen (VCA) and Epstein–Barr nuclear antigen (EBNA) are commonly used to help determine the stage of infection. In the setting of immunosuppression, HIV infection, or other immunodeficiency states, the risks of EBV reactivation and EBV-associated diseases are significantly increased (6). In addition, EBV can be classified into type 1 and type 2, mainly based on sequence polymorphisms in the EBNA2 and EBNA3 genes (7).

In addition to its association with benign diseases, such as infectious mononucleosis, EBV is also involved in the development of various rheumatic and immune-mediated diseases as well as malignancies. In particular, it has a well-established or highly suggestive association with multiple lymphoid malignancies and epithelial-derived tumors, including nasopharyngeal carcinoma and certain gastrointestinal tumors (8). Recent studies have also detected EBV DNA in nasal polyps and intestinal mucosal/polypoid lesions, suggesting that EBV may be involved in certain focal mucosal inflammatory or hyperplastic changes (9–12). However, reports of EBV presenting as an “inflammatory polyp/inflammatory polypoid lesion within the tracheal lumen” and causing progressive airway obstruction are extremely rare. Moreover, histological differentiation from EBV-associated lymphoproliferative disorders or other neoplastic lesions may be challenging, which can lead to diagnostic bias or misdiagnosis (1, 2).

In recent years, targeted next-generation sequencing (tNGS) has gradually become an important adjunctive tool for the etiological diagnosis of complex infections. By using strategies such as highly multiplexed PCR or probe capture to pre-enrich nucleic acids from specific pathogens prior to sequencing, tNGS offers several advantages over untargeted metagenomic next-generation sequencing in selected clinical scenarios, including higher sensitivity for targeted pathogens, reduced interference from host background nucleic acids, lower sequencing data requirements, lower testing costs, and shorter turnaround time (13–15). In particular, when conventional microbiological tests are negative, or when histopathology suggests inflammation but lacks definitive etiological evidence, tNGS can provide direct molecular evidence of pathogens in tissue samples (16). Therefore, for such rare and atypical EBV-associated airway lesions, obtaining sufficient tissue samples and integrating histopathology, immunohistochemistry, EBER *in situ* hybridization, and tissue-based tNGS may help improve diagnostic accuracy and avoid misdiagnosis.

## Case presentation

**Figure 1:**
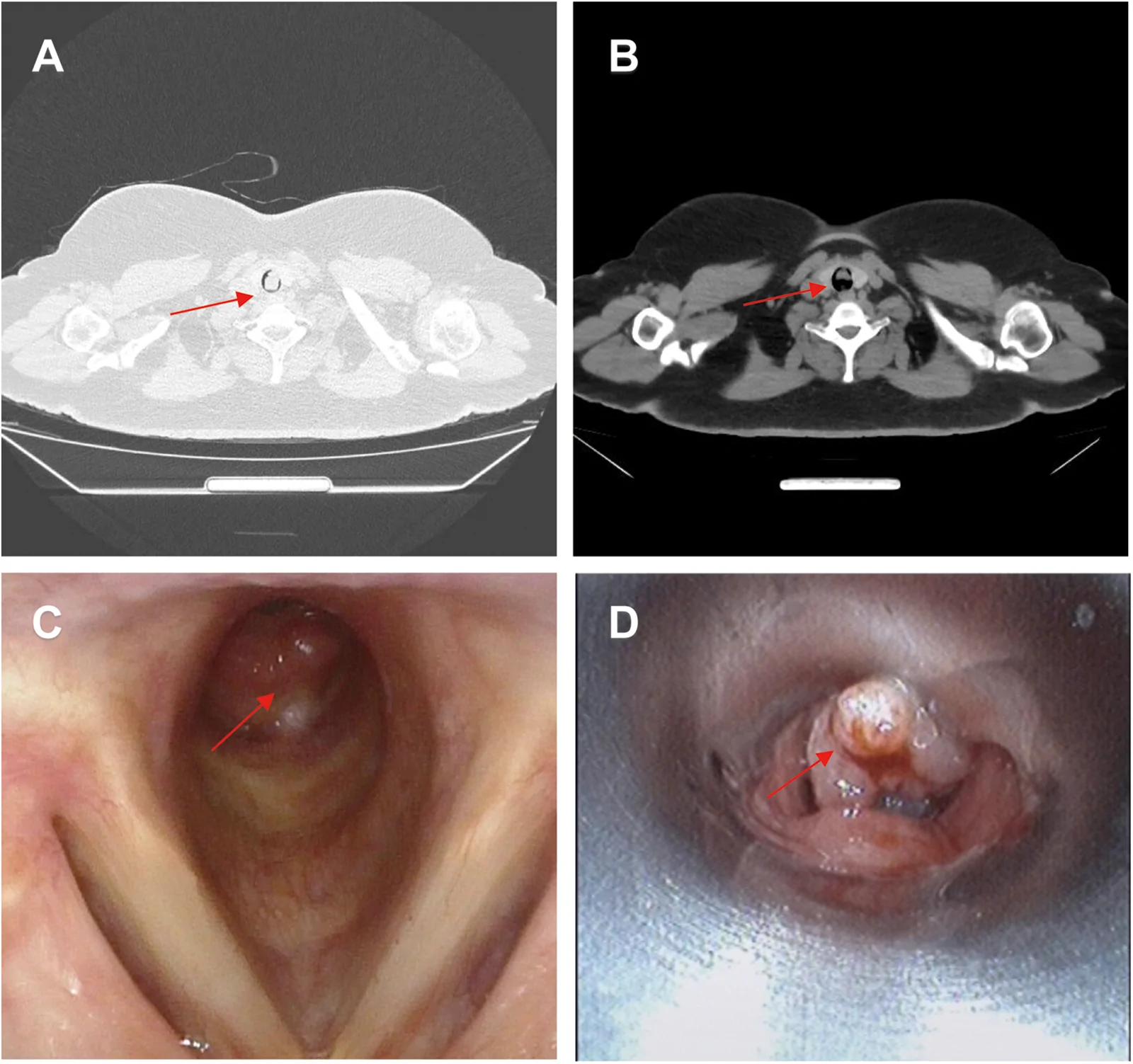
CT and endoscopic findings of the proximal cervical tracheal lesion. **(A,B)** Axial computed tomography images showing a high-density intraluminal lesion arising from the left anterior wall of the proximal cervical trachea, resulting in marked luminal narrowing. The red arrows indicate the lesion; **(C)** Flexible laryngoscopic view showing the laryngeal inlet and a polypoid lesion visible distal to the vocal cords, with no definite involvement of the true subglottic mucosa; **(D)** Rigid bronchoscopic view showing a cauliflower-like polypoid lesion within the proximal cervical tracheal lumen before cryoresection.

**Figure 2:**
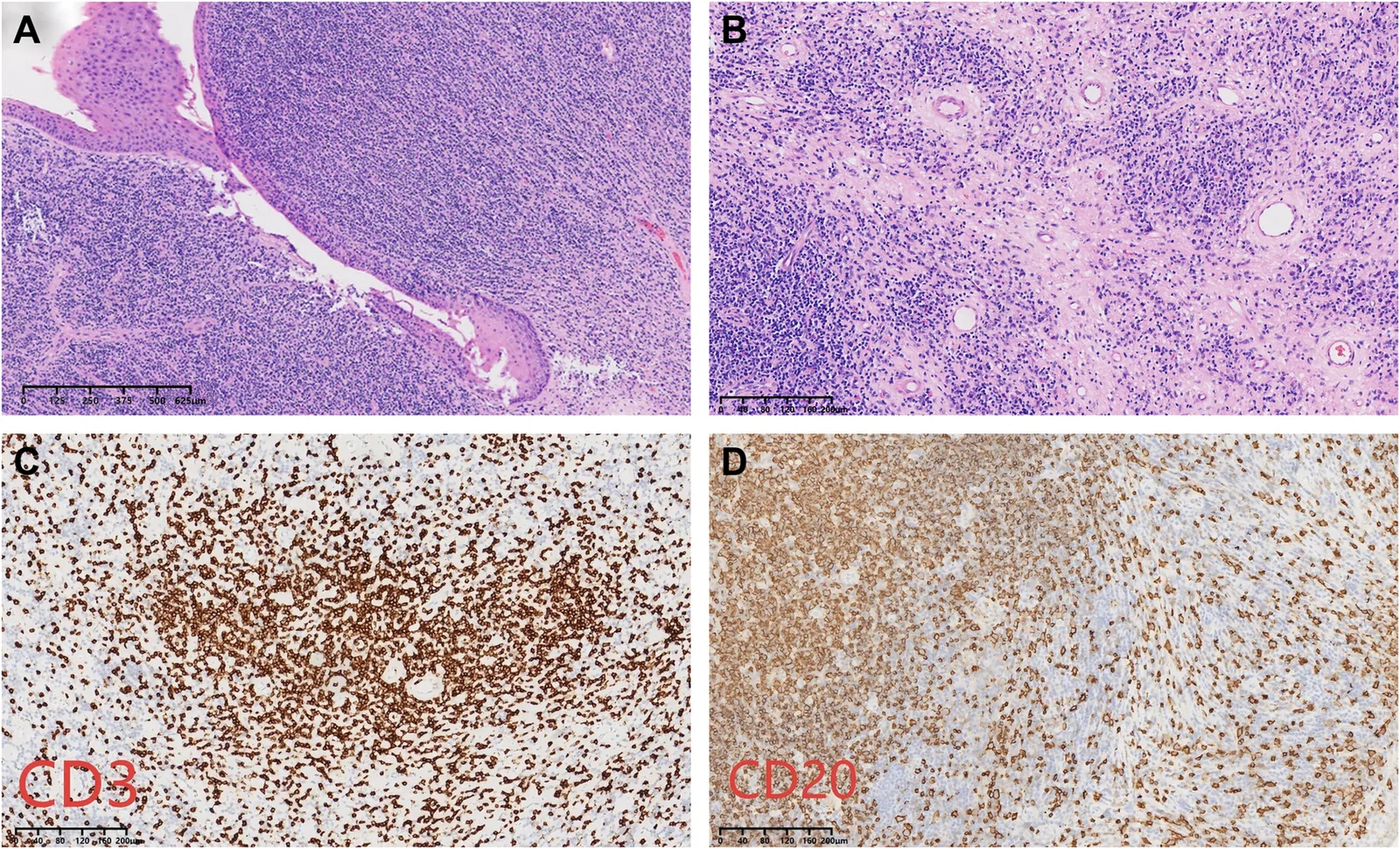
Histopathological and immunohistochemical findings of the proximal cervical tracheal lesion. **(A)** Low-power hematoxylin and eosin staining shows polypoid tissue fragments from the proximal cervical trachea, with dense lymphoid proliferation beneath the squamous epithelium. Objective magnification, ×4; scale bar, 625 μm. **(B)** Higher-power hematoxylin and eosin staining demonstrates subepithelial reactive lymphoid proliferation with fibrous stroma and small vessels. Objective magnification, ×10; scale bar, 200 μm. **(C)** CD3 immunohistochemistry highlights abundant T lymphocytes within the reactive lymphoid infiltrate. Objective magnification, ×10; scale bar, 200 μm. **(D)** CD20 immunohistochemistry highlights B lymphocytes, showing a mixed T- and B-cell lymphoid population, consistent with reactive lymphoid hyperplasia. Objective magnification, ×10; scale bar, 200 μm.

A 35-year-old female presented with a 1-month history of progressive dyspnea, accompanied with wheezing, leading to admission on day 0. Post-admission, her dyspnea worsened, with significant shortness of breath and stridor upon minimal exertion. She denied fever, chest pain, nausea, vomiting, night sweats, or weight loss. She had no prior history of recurrent respiratory infections, and her family history was unremarkable. Additional history was reviewed to evaluate possible HPV-associated recurrent respiratory papillomatosis and other causes of reactive tracheal lesions. The patient had no previous history of HPV infection, cervical dysplasia, cervical or vulvar intraepithelial neoplasia, anogenital condylomata, or other HPV-related disease. She had not received HPV vaccination. She also had no history of juvenile-onset respiratory papillomatosis, airway papillomatosis at any site, or previous treatment for airway papillomatosis. No relevant obstetric history related to respiratory papillomatosis was identified. The patient had no history of endotracheal intubation, airway stent implantation, tracheostomy, aspiration pneumonia, or gastro-oesophageal reflux disease. She was considered immunocompetent, with no known immunodeficiency, organ transplantation, hematological malignancy, long-term corticosteroid use, or other immunosuppressive therapy. Physical examination revealed jugular venous distension, evident suprasternal retractions, coarse breath sounds bilaterally with audible wheezing, regular heart rhythm without murmurs, and no palpable hepatosplenomegaly or lymphadenopathy. Laboratory findings included a white blood cell count of 8.20 × 10^9/L, with neutrophils at 70.9%, lymphocytes at 22.9%, and monocytes at 5.3%. High-sensitivity C-reactive protein was 12.36 mg/L. Arterial blood gas analysis (on 2 L/min oxygen) showed a pH of 7.38, PaCO₂ of 45.2 mmHg, and PaO₂ of 131.0 mmHg. Tumor markers, biochemical parameters, and LDH were within normal limits. Urgent flexible laryngoscopy and contrast-enhanced chest CT revealed a solitary intraluminal polypoid lesion located in the proximal cervical trachea rather than the true subglottic region. Multiplanar CT reconstruction showed that the lesion arose from the left anterior wall of the proximal cervical trachea, with no subglottic involvement and no crossing of the subglottic region and trachea. The proximal margin of the lesion was approximately 26.3 mm caudal to the vocal cords, and the distal margin was approximately 40.8 mm caudal to the vocal cords, giving a craniocaudal length of approximately 14.5 mm. The distal margin of the lesion was approximately 9.34 cm from the carina. The minimum residual airway diameter at the narrowest point was approximately 1.7 mm, compared with an adjacent normal tracheal diameter of approximately 16.1 mm. The adjacent normal tracheal luminal area was approximately 2.63 cm², and the residual luminal area at the narrowest point was approximately 1.15 cm²; the estimated luminal obstruction was approximately 54% (Table 1 and Supplementary Figure S1). Axial CT images and endoscopic views further demonstrated the intraluminal polypoid morphology of the lesion and its relationship to the proximal cervical tracheal lumen (Figure 1). A multidisciplinary team (MDT) discussion involving otolaryngology, critical care respiratory medicine, and radiology concluded that relieving the airway obstruction was the most critical issue. Consequently, emergency resection of the tracheal lesion via rigid bronchoscopy was performed on day 0. Bronchoscopic findings confirmed a cauliflower-like polypoid lesion in the proximal cervical trachea, immediately distal to the laryngeal inlet, without definite involvement of the subglottic mucosa. The lesion was removed via cryoresection (Figure 1D). The specimen was submitted for histopathological and immunohistochemical examination. Initial pathology suggested multiple mucosal polyps and reactive lymphoid hyperplasia. Immunohistochemistry revealed CD3 (T-cells+), CD20 (B-cells), CD21 (follicular dendritic cells+), CD138 (plasma cells+), Ki-67 (focal+), CD38 (focal+), CD43 (B-cells+), Desmin (stromal cells), SMA (stromal and vascular endothelium+), and Cyclin D1 (scattered+). Representative histopathological and immunohistochemical findings are shown in Figure 2. In conjunction with morphology and immunophenotype, the findings favored benign polyps secondary to chronic inflammatory stimulation, with no evidence of malignancy. p16 immunohistochemistry was not performed, and no dedicated HPV DNA/RNA testing was available in this case. Therefore, HPV-associated recurrent respiratory papillomatosis could not be completely excluded at the molecular level. However, the available clinical history did not support previous HPV infection, HPV-related disease, or airway papillomatosis, and the pathological interpretation favored a reactive inflammatory polypoid process rather than a papillomatous epithelial proliferation. Evidence supporting tuberculosis, Langerhans cell histiocytosis, or tracheal amyloidosis was also absent.

**Table 1:** Imaging characteristics and quantitative assessment of the tracheal lesion.

Parameter	Finding
Number of lesions	Solitary
Anatomical site	Proximal cervical trachea
Site of origin	Left anterior wall of the proximal cervical trachea
Subglottic involvement	No
Involvement of proximal cervical trachea	Yes
Extension from the subglottic region to the proximal cervical trachea	No
Crossing of the subglottic region and trachea	No
Proximal margin of the lesion	Approximately 26.3 mm caudal to the vocal cords
Distal margin of the lesion	Approximately 40.8 mm caudal to the vocal cords
Craniocaudal length	Approximately 14.5 mm
Distance from the distal margin to the carina	Approximately 9.34 cm
Degree of airway obstruction	Severe
Minimum residual airway diameter at the narrowest point	Approximately 1.7 mm
Adjacent normal tracheal diameter	Approximately 16.1 mm
Adjacent normal tracheal luminal area	Approximately 2.63 cm²
Residual luminal area at the narrowest point	Approximately 1.15 cm²
Estimated luminal obstruction	Approximately 54%

On day 4 after admission, corresponding to postoperative day 4, repeat bronchoscopy showed a residual polyp stump arising from the left anterior wall of the proximal cervical trachea and protruding into the tracheal lumen, while the tracheal lumen remained patent. Endoscopic cryoablation was performed at the base of the residual lesion, with a freeze–thaw duration of 90 s per cycle for three cycles. No bleeding was observed after the procedure. The specimens were submitted to a tertiary referral hospital for pathological consultation. The consultation diagnosis was polypoid tissue fragments from the proximal cervical trachea, showing reactive lymphoid hyperplasia beneath the squamous epithelium. EBER *in situ* hybridization demonstrated positivity in a subset of lymphocytes, suggestive of Epstein–Barr virus (EBV) infection, with no evidence of lymphoma. Following treatment, the patient's chest tightness and dyspnea improved markedly, wheezing resolved, and she was discharged on day 5 after admission. Further immunohistochemical results became available on day 18 after admission, as follows: CD3, CD5, and CD43 were positive in T cells; CD21 and CD23 highlighted the follicular dendritic cell network; CD10 and Bcl-6 were positive in germinal centers; Bcl-2 was negative in germinal centers; Kappa and Lambda were positive in a subset of plasma cells; MNDA was partially positive; CD138 was positive in plasma cells; PAX-5 was positive in B cells; Ki-67 showed approximately 20% positivity in the interfollicular areas; Cyclin D1 was negative; and EBER was focally positive, with approximately 100 positive cells per high-power field. Serological testing performed on day 23 after admission further demonstrated positivity for EBV infection-related antibodies, including EBV nuclear antigen IgG (EBV-NA IgG) at 179.86 and EBV viral capsid antigen IgG (EBV-CA IgG) at 43.31. Taken together, the clinical, histopathological, and immunophenotypic findings indicated that the EBER-positive cells were mainly located within the subepithelial reactive lymphoid tissue, while proliferative activity was predominantly confined to the interfollicular areas composed of T lymphocytes and histiocytes. No vascular invasion or coagulative necrosis was identified. Overall, these findings favored an infection-related reactive process rather than lymphoma.

**Figure 3:**
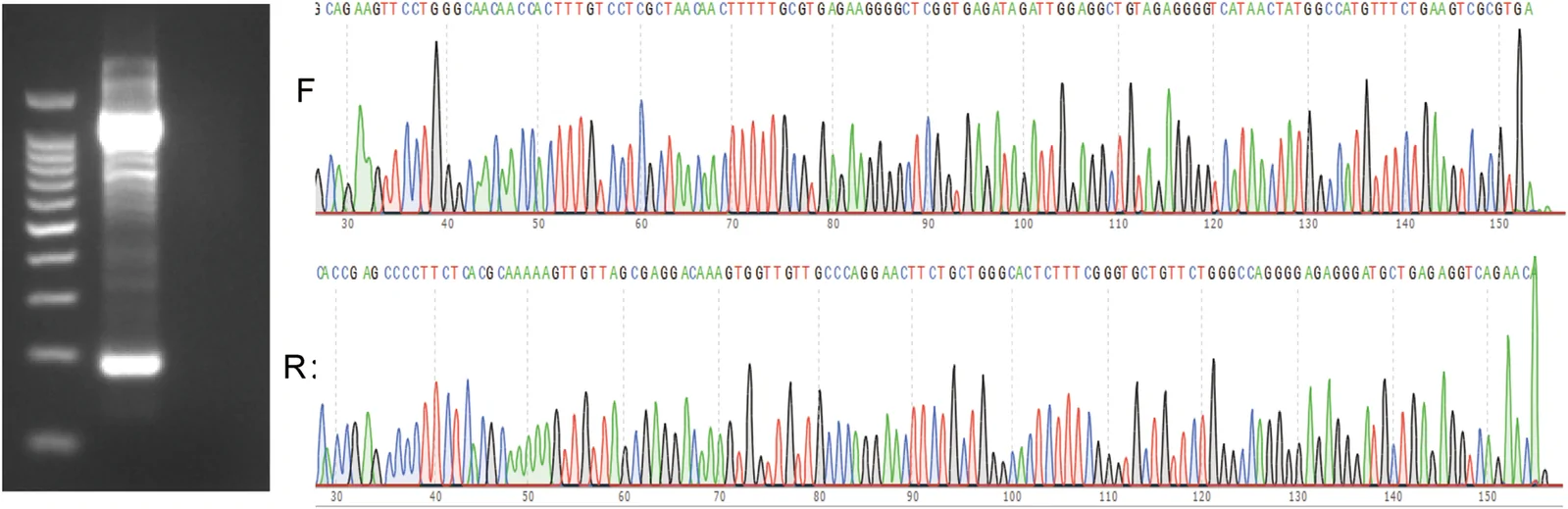
Molecular detection and sequencing confirmation of EBV.

**Figure 4:**
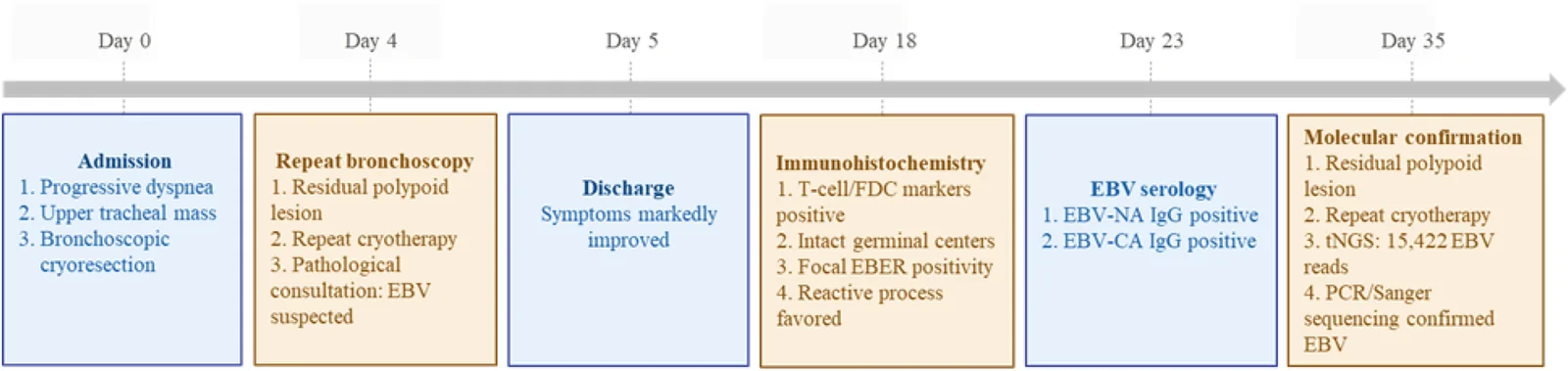
Clinical course and diagnostic timeline. The timeline summarizes the patient's clinical presentation, bronchoscopic interventions, pathological consultation, EBV serology, tissue-based targeted next-generation sequencing, and molecular validation. The day of admission was defined as day 0. Emergency rigid bronchoscopic cryoresection was performed on day 0; repeat bronchoscopy and cryotherapy were performed on day 4 after admission/postoperative day 4; discharge occurred on day 5; additional immunohistochemical results became available on day 18; EBV serology was performed on day 23; and follow-up bronchoscopy with tissue-based tNGS was performed on day 35. After day 35, the patient did not continue follow-up at our institution, and follow-up was interrupted.

On day 35 after admission, follow-up bronchoscopy again showed a residual polyp stump arising from the left anterior wall of the proximal cervical trachea. The residual lesion appeared as a localized elevation with partial scar formation, and the basal extent of the lesion was smaller than that observed during the previous bronchoscopy. The mucosal surface was continuous and smooth, and the tracheal lumen remained patent. Endoscopic cryoablation was again performed at the base of the residual lesion, with a freeze–thaw duration of 90 s per cycle for three cycles. No bleeding was observed after the procedure. Tissue-based tNGS detected 15,422 Epstein–Barr virus (EBV) sequence reads. EBV-specific PCR followed by Sanger sequencing further confirmed the presence of EBV in the tissue sample. The primers used for EBV amplification are listed in Table 2. Representative agarose gel electrophoresis and Sanger sequencing chromatograms are shown in Figure 3. The obtained consensus sequence was further analyzed by BLASTn, and the BLASTn results are summarized in Table 3. The clinical course and diagnostic timeline of the patient are summarized in Figure 4. Integrating clinical presentation, imaging and endoscopic findings, histopathology and immunohistochemistry, EBER *in situ* hybridization, and tissue tNGS with molecular validation, the final diagnosis was established as Epstein–Barr virus-associated inflammatory polyp of the proximal cervical trachea. After the bronchoscopic treatment on day 35 after admission, the patient did not return to our institution for further treatment or scheduled follow-up. Therefore, follow-up at our institution was interrupted. As of manuscript submission, 300 days after the last documented clinical event, no additional clinical, bronchoscopic, serological, or molecular follow-up data were available.

**Table 2:** Specific primers for *Epstein–Barr virus.*

Primer name	Sequence	Product length
DV00715-F	GTTCTGACCTCTCAGCATCCC	185bp
DV00715-R	CACGCGACTTCAGAAACATGG	

**Table 3:** BLASTn analysis of the Sanger sequencing product.

PCR product	Length	Closest BLASTn match	Accession no.	Query coverage	Identity	E-value
EBV target fragment	342 bp	Human gammaherpesvirus 4 UPN138 tumor DNA, nearly complete genome	AP019067.1	99%	100.00%	1.00E-89
EBV target fragment	342 bp	Human gammaherpesvirus 4 isolate HKNPC62, partial genome	MH590573.1	99%	100.00%	1.00E-89
EBV target fragment	342 bp	Human gammaherpesvirus 4 genome assembly, plasmid: 1	LR994533.1	99%	100.00%	1.00E-89
EBV target fragment	342 bp	Human gammaherpesvirus 4 isolate HKHD85, partial genome	MH590454.1	99%	100.00%	1.00E-89
EBV target fragment	342 bp	Human gammaherpesvirus 4 strain VGO, partial genome	KP682960.1	99%	100.00%	1.00E-89

## Discussion

The salient feature of this case is that an immunocompetent young woman presented with progressive dyspnea and stridor, with imaging and endoscopic examinations revealing a solitary intraluminal polypoid lesion arising from the left anterior wall of the proximal cervical trachea, rather than from the true subglottic region. The lesion extended from approximately 26.3 mm to 40.8 mm caudal to the vocal cords, with a craniocaudal length of approximately 14.5 mm, and caused clinically significant central airway obstruction. The minimum residual airway diameter at the narrowest point was approximately 1.7 mm, and the estimated luminal obstruction was approximately 54%. After urgent relief of obstruction by cryoresection and cryoablation via rigid bronchoscopy, histopathological examination demonstrated reactive lymphoid hyperplasia beneath the squamous epithelium, with scattered EBER positivity by *in situ* hybridization. Combined with the detection of high-abundance Epstein–Barr virus (EBV) sequences in tissue by targeted metagenomic next-generation sequencing (tNGS), further validated by PCR/Sanger sequencing, the final diagnosis was established as an EBV infection-associated inflammatory polyp of the proximal cervical trachea. Because space-occupying lesions of the large airways are predominantly malignant, whereas inflammatory, infectious, and granulomatous lesions constitute an important proportion of benign entities, it is crucial to obtain sufficient tissue for stratified histopathological and etiological evaluation while urgently relieving central airway obstruction (1).

The differential diagnosis of a polypoid or cauliflower-like lesion within the adult tracheal lumen is broad and should include HPV-associated recurrent respiratory papillomatosis, primary tracheal neoplasms, inflammatory myofibroblastic tumor, specific infectious granulomas such as tuberculosis or fungal infection, and airway involvement by systemic diseases including IgG4-related disease and amyloidosis (17). HPV-associated recurrent respiratory papillomatosis is particularly important in the differential diagnosis of cauliflower-like airway lesions, especially when lesions are multifocal, persistent, or recurrent after debulking. In the present case, the lesion was confirmed radiologically and bronchoscopically to be located in the proximal cervical trachea rather than the true subglottic region. The patient had no previous history of HPV infection, cervical dysplasia, cervical or vulvar intraepithelial neoplasia, anogenital condylomata, HPV-related disease, juvenile-onset respiratory papillomatosis, airway papillomatosis at any site, or previous treatment for airway papillomatosis. She had not received HPV vaccination. She also had no history of endotracheal intubation, airway stent implantation, tracheostomy, aspiration pneumonia, or gastro-oesophageal reflux disease. The patient was immunocompetent, with no known immunodeficiency or immunosuppressive condition. However, p16 immunohistochemistry was not performed, and no dedicated HPV DNA/RNA testing was available. Therefore, HPV-associated recurrent respiratory papillomatosis could not be completely excluded at the molecular level. This represents a limitation of the present case, and p16 immunohistochemistry together with HPV testing should be performed if recurrent or residual lesions are sampled during follow-up.

When tracheal polypoid lesions are accompanied by submucosal lymphoid hyperplasia and EBER positivity, the diagnostic focus should also shift toward distinguishing infection- or inflammation-driven reactive hyperplasia from EBV-associated lymphoproliferative disorders or lymphoma. The 2022 WHO classification emphasizes that EBV-associated lesions represent a disease spectrum, and diagnosis requires comprehensive assessment of architectural destruction, clonality, aberrant immunophenotype, proliferative activity, necrosis, vascular invasion, and the relevant clinical context; a neoplastic diagnosis should not be made on the basis of EBER positivity alone (18). In the present case, repeated tissue sampling consistently showed preserved follicular dendritic cell networks, as highlighted by CD21 and CD23, CD10 and Bcl-6 positivity with Bcl-2 negativity in germinal centers, polytypic plasma cell light-chain expression, a low Ki-67 proliferation index predominantly in the interfollicular areas, and no evidence of necrosis or vascular invasion. Taken together, these findings favored reactive follicular hyperplasia or infection-related changes rather than lymphoma.

Regarding the potential pathogenesis, polypoid lesions essentially represent focal mucosal tissue hyperplasia and usually arise in the setting of repeated cycles of “injury–inflammation–repair.” Persistent or recurrent inflammation can promote immune-cell aggregation and the release of multiple inflammatory mediators, such as TNF-α and IL-6, as well as reactive oxygen species (ROS), leading to sustained mucosal barrier injury and aberrant repair. These processes may ultimately drive local tissue overgrowth and the formation of polypoid protrusions. EBV has well-defined cellular tropism and entry mechanisms. It can attach to B cells through the interaction between gp350/220 and CD21/CD35, and viral entry is further facilitated by molecules such as gp42 and HLA class II. In epithelial cells lacking CD21, EBV entry is more likely mediated by glycoproteins such as gH/gL. The early host response to EBV infection may be accompanied by upregulation of pro-inflammatory cytokines and activation of inflammation-related signaling pathways, including NF-*κ*B and STAT (19–21). Previous studies have shown active viral replication and a strong inflammatory response in EBV-infected proximal gastric regions, including the cardia, fundus, and body (22), suggesting that EBV infection may be associated with persistent inflammation. During latency, latent proteins such as LMP1 and LMP2 may also contribute to the maintenance of a chronic inflammatory microenvironment and reactive tissue hyperplasia through sustained activation of NF-*κ*B, JAK/STAT, and MAPK/ERK signaling pathways (23, 24). Therefore, within a specific local microenvironment, EBV may participate in the development of polypoid changes by amplifying local inflammation and modulating mucosal repair. Previous reports of EBV-associated inflammatory lesions involving the respiratory tract are extremely limited. Wang et al. reported a rare case of EBV-associated inflammatory granuloma of the trachea, in which EBV infection was identified in granulomatous tracheal tissue (25). However, the previously reported lesion was characterized predominantly by granulomatous inflammation, whereas the present case exhibited a solitary intraluminal polypoid lesion arising from the proximal cervical trachea and causing clinically significant central airway obstruction. Histologically, our case showed reactive lymphoid hyperplasia beneath the squamous epithelium rather than granulomatous inflammation. Moreover, tissue-based tNGS detected abundant EBV sequences in the lesion, which were further confirmed by PCR and Sanger sequencing, providing additional molecular evidence supporting the association between EBV infection and the localized tracheal lesion. The diagnosis of EBV-associated inflammatory tracheal polyp in this case was established through an integrated and mutually corroborative diagnostic framework, including endoscopic morphology, histopathological evaluation, immunophenotypic analysis, EBER *in situ* hybridization, and molecular pathogen detection. When tissue morphology indicates only reactive hyperplasia and EBER-positive cells are scattered, a single diagnostic approach may be insufficient to establish a reliable association between pathogen infection and tissue lesions. In this context, tNGS provided important diagnostic support by identifying high-abundance EBV nucleic acid signals within the lesion tissue, thereby reducing uncertainty caused by incidental detection or potential contamination. This evidence framework expands the spectrum of EBV-associated inflammatory airway lesions and highlights the importance of combining pathological and molecular approaches in the evaluation of rare EBV-related airway disorders.

In recent years, clinical pathogen sequencing technologies have demonstrated unique value in difficult-to-diagnose infections, infections occurring at atypical anatomical sites, and cases in which conventional microbiological tests are negative but infection remains clinically suspected. Particularly for tissue specimens, pathogen sequencing can provide direct molecular evidence of microbial involvement and complement conventional histopathological assessment, thereby improving the identification of pathogen-associated inflammatory lesions (16).

Nevertheless, EBV-associated hyperplastic lesions should still be carefully distinguished from chronic active EBV disease (CAEBV). CAEBV is a rare but severe EBV-associated disease spectrum that commonly presents with persistent or recurrent infectious mononucleosis-like symptoms, such as prolonged fever, hepatosplenomegaly, and lymphadenopathy, and may involve multiple organs. Some patients may progress to EBV-associated T/NK-cell lymphoproliferative disorders, for which therapeutic strategies and prognosis differ substantially from those of localized reactive lesions (26). In the present case, the patient had no persistent or recurrent fever, hepatosplenomegaly, or peripheral lymphadenopathy, and her complete blood count and LDH level were essentially normal. Serological testing did not show a typical acute infection profile, such as VCA-IgM positivity, and pathological examination revealed no evidence of neoplastic architectural destruction or monoclonality. Therefore, the possibility of CAEBV was considered relatively low. However, given the critical airway location of the lesion and the risks of residual disease and recurrence, risk stratification and dynamic monitoring during follow-up are recommended. These should include periodic bronchoscopy to evaluate recurrence and cicatricial stenosis, as well as monitoring of peripheral blood EBV DNA load, complete blood count, liver function, and LDH. If fever, hepatosplenomegaly, lymphadenopathy, cytopenia, or persistently elevated peripheral blood EBV DNA occurs, prompt referral to infectious disease or hematology specialists is warranted for further evaluation of CAEBV and EBV-associated T/NK-cell lymphoproliferative disorders (27).

However, in the present case, follow-up was limited. Residual polypoid tissue was observed at both documented follow-up bronchoscopies. On day 4 after admission, corresponding to postoperative day 4, bronchoscopy showed a residual polyp stump at the left anterior wall of the proximal cervical trachea protruding into the lumen, although the trachea remained patent; cryoablation was performed at the lesion base for 90 s per cycle for three cycles, without post-procedural bleeding. On day 35 after admission, corresponding to postoperative day 35, bronchoscopy again showed a residual polyp stump at the same site, with local elevation, partial scar formation, a smaller basal extent than before, and a continuous and smooth mucosal surface; repeat cryoablation was performed using the same freeze–thaw protocol, again without bleeding. After the day-35 bronchoscopic cryotherapy and molecular confirmation of EBV in the tracheal tissue, the patient did not continue treatment or scheduled follow-up at our institution. Follow-up was therefore interrupted, and no further in-hospital bronchoscopic, serological, imaging, or molecular data were available up to the time of manuscript submission. The last documented clinical event occurred on day 35 after admission, and the manuscript was submitted 300 days after this event. This limited follow-up is an important limitation of the present report. Another limitation is the absence of p16 immunohistochemistry and dedicated HPV molecular testing in the available tissue specimens. Although the clinical history and pathological interpretation did not support HPV-associated recurrent respiratory papillomatosis, HPV-associated disease could not be completely excluded at the molecular level. Further p16 immunohistochemistry and HPV testing will be considered if recurrent or residual lesions are sampled during follow-up.

## Data Availability

The data that support the findings of this study are available from the corresponding author upon reasonable request.
