# Supplementary material for: Epstein–Barr virus-associated inflammatory polyp of the proximal cervical trachea causing central airway obstruction: a case report
Source: Front Med (Lausanne). 2026 Sep 3;13:1940485. doi: 10.3389/fmed.2026.1940485 (PMC13581744; doi:10.3389/fmed.2026.1940485)

Supplementary Figure 1. CT-based anatomical localization and quantitative assessment of the tracheal lesion. (A) Sagittal multiplanar reconstruction showing the proximal and distal margins of the intraluminal lesion in relation to the vocal cords. The lesion was located in the proximal cervical trachea, with its proximal margin approximately 26.3 mm caudal to the vocal cords and its distal margin approximately 40.8 mm caudal to the vocal cords. The craniocaudal length was approximately 14.5 mm. (B) Coronal reconstruction showing the distance from the distal margin of the lesion to the carina, approximately 9.34 cm. © Sagittal image showing the minimum residual airway diameter at the narrowest point, approximately 1.7 mm, compared with the adjacent normal tracheal diameter of approximately 16.1 mm. (D) Axial CT image at the level of maximal obstruction showing severe narrowing of the tracheal lumen. (E) Axial CT image of the adjacent normal trachea used as the reference level for luminal measurement. The adjacent normal tracheal luminal area was approximately 2.63 cm², the residual luminal area at the narrowest point was approximately 1.15 cm², and the estimated luminal obstruction was approximately 54%. The lesion arose from the left anterior wall of the proximal cervical trachea, without true subglottic involvement.

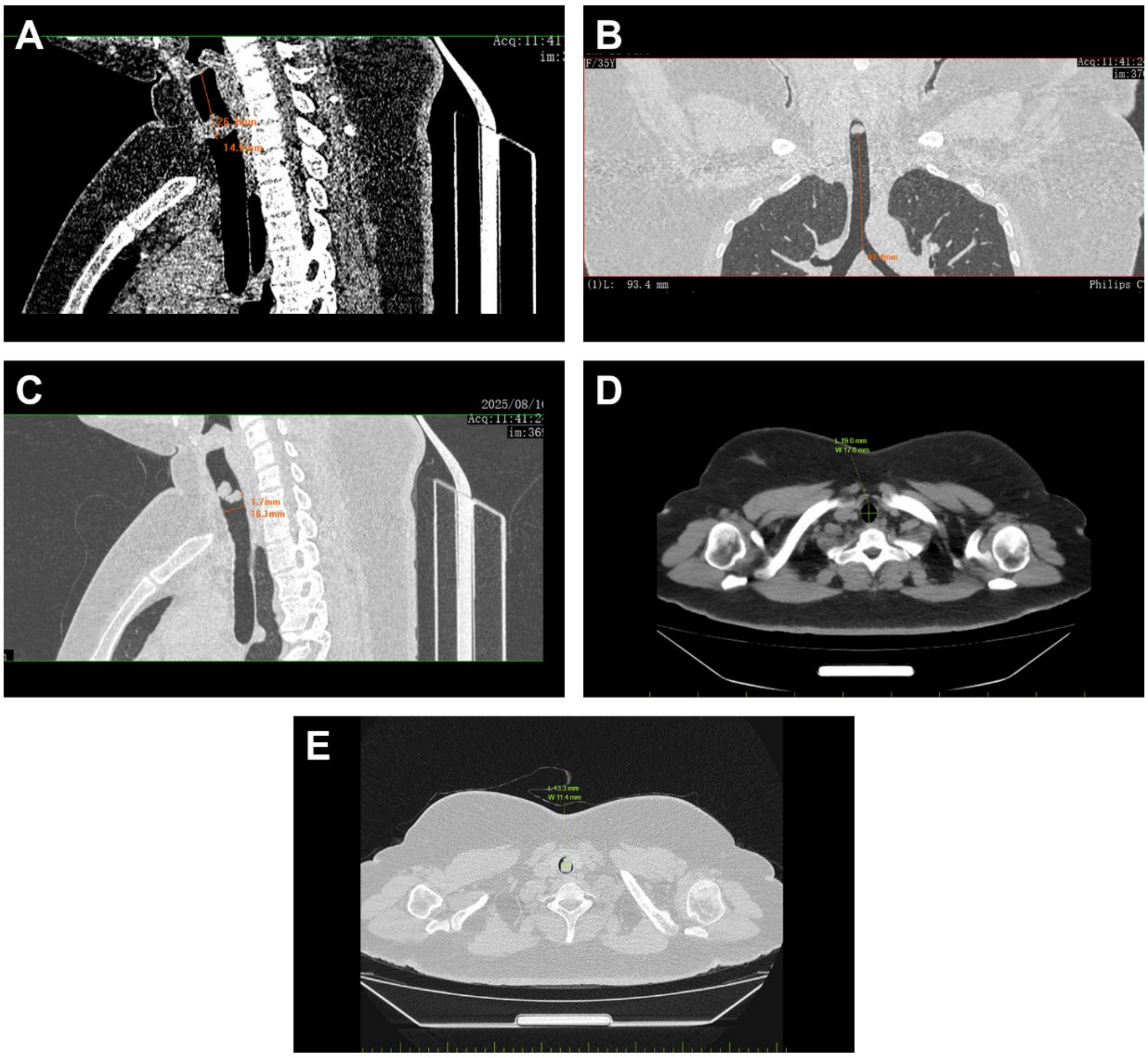

Supplement: Supplementary file 1 [file Image1.pdf]
